# Evaluation of Ki-67 proliferation and apoptotic index before, during and after neoadjuvant chemotherapy for primary breast cancer

**DOI:** 10.1186/bcr1508

**Published:** 2006-06-21

**Authors:** Russell Burcombe, George D Wilson, Mitch Dowsett, Ifty Khan, Paul I Richman, Frances Daley, Simone Detre, Andreas Makris

**Affiliations:** 1 Kent Oncology Centre, Maidstone Hospital, Hermitage Lane, Maidstone, Kent ME16 9QQ, UK; 2 Department of Radiation Biology, William Beaumont Hospital, 3811 W. Thirteen Mile Road, 105-R1, Royal Oak, MI 48073, USA; 3 Institute of Cancer Research, Royal Marsden Hospital, Fulham Road, London SW3 6JJ UK; 4 The Royal London Hospital, Whitechapel, London E1 1BB UK; 5 Mount Vernon Hospital, Rickmansworth Road, Northwood, Middlesex HA6 2RN, UK

## Abstract

**Introduction:**

Biological markers that reliably predict clinical or pathological response to primary systemic therapy early during a course of chemotherapy may have considerable clinical potential. This study evaluated changes in Ki-67 labeling index and apoptotic index (AI) before, during, and after neoadjuvant anthracycline chemotherapy.

**Methods:**

Twenty-seven patients receiving neoadjuvant FEC (5-fluorouracil, epirubicin, and cyclophosphamide) chemotherapy for operable breast cancer underwent repeat core biopsy after 21 days of treatment. Tissue from pre-treatment biopsy, day 21 and surgery was analysed for Ki-67 index and AI.

**Results:**

The objective clinical response rate was 56%. Eight patients (31%) achieved a pathological response by histological criteria; two patients had a near-complete pathological response. A reduction in Ki-67 index was observed in 68% of patients at day 21 and 72% at surgery; Ki-67 index increased between day 21 and surgery in 54%. AI decreased in 50% of tumours by day 21, increased in 45% and was unchanged in one patient; 56% demonstrated rebound increases in AI by the time of surgery. Neither pre-treatment nor post-chemotherapy median Ki-67 index nor median AI at all three time points or relative changes at day 21 and surgery differed significantly between clinical or pathological responders and non-responders. Clinical responders had lower median Ki-67 indices at day 21 (11.4% versus 27.0%, *p *= 0.02) and significantly greater percentage reductions in Ki-67 at day 21 than did non-responders (-50.6% versus -5.3%, *p *= 0.04). The median day-21 Ki-67 was higher in pathological responders (30.3% versus 14.1%, *p *= 0.046). A trend toward increased AI at day 21 in pathological responders was observed (5.30 versus 1.68, *p *= 0.12). Increased day-21 AI was a statistically significant predictor of pathological response (*p *= 0.049). A strong trend for predicting pathological response was seen with higher Ki-67 indices at day 21 and AI at surgery (*p *= 0.06 and 0.06, respectively).

**Conclusion:**

The clinical utility of early changes in biological marker expression during chemotherapy remains unclear. Until further prospectively validated evidence confirming the reliability of predictive markers is available, clinical decision-making should not be based upon individual biological tumour marker profiles.

## Introduction

Primary breast carcinomas treated with neoadjuvant chemotherapy or primary systemic therapy (PST) provide an ideal model to evaluate the role of biological markers as predictive and prognostic factors. Many retrospective studies have identified patterns of biomarker expression before or after chemotherapy which have predictive or prognostic significance in relation to different clinical endpoints. However, no single pre-treatment marker that can accurately predict response to PST has been found to be of clinical utility to date. Despite high objective response rates to PST, a small proportion of patients will fail to respond or will progress during primary chemotherapy. The early identification of non-responders may spare these patients the unnecessary toxicity of ineffective chemotherapy and allow them to be offered alternative treatment strategies or non-cross-resistant regimens. Biological markers that can reliably predict clinical or pathological response early during a course of treatment therefore have considerable clinical potential.

In randomised clinical trials, PST confers equivalent survival and increased breast conservation rates compared with primary surgery and adjuvant cytotoxic chemotherapy [1,2]. Complete pathological response (pCR) is a strong prognostic indicator for prolonged disease-free and overall survival [3]. Patients achieving a complete clinical response (cCR) also have a statistically superior disease-free and overall survival advantage over clinical non-responders [3,4]. It should be acknowledged that in the smaller of these two studies [4], patients received some chemotherapy post-operatively. Clinical response is frequently used as a surrogate intermediate endpoint for predicting disease-free survival and outcome after primary chemotherapy; pCR is a valid intermediate surrogate endpoint for predicting overall survival.

The ability to biopsy breast tumours *in situ *during primary chemotherapy provides a unique opportunity to evaluate molecular markers in the tumour before and during treatment and to relate these changes to both clinical and pathological response. Immunohistochemical analysis of tumour material from repeat biopsies during treatment may therefore help unravel the complex molecular mechanisms that ultimately determine clinical outcomes and thereby provide more useful and reliable intermediate predictive and prognostic factors.

The nuclear antigen Ki-67 is a proliferation marker expressed only in cycling cells. A strong correlation between S-phase fraction and Ki-67 index has been demonstrated [5-7]. Consequently, quantitative assessment of Ki-67 staining on paraffin-embedded tumour sections provides an accurate estimate of the proliferation index of individual tumours. Cytotoxic chemotherapy induces programmed cell death by apoptosis. The percentage of apoptotic cells in tumour sections may be measured by labeling fragmented DNA breaks and calculating the apoptotic index (AI) using the TUNEL (terminal transferase uridyl nick-end labeling) assay [8].

In this study, Ki-67 and apoptosis were assessed on histological material before, during, and after PST for operable breast cancer to evaluate whether early changes in proliferation or apoptosis predict clinical or pathological response to treatment.

## Materials and methods

### Treatment protocol

A series of 39 female patients with operable (T2–T4, N0 or N1, M0) invasive primary breast carcinoma were identified between May 1999 and July 2001. Patients with metastatic disease (M1) or inflammatory breast cancer (T4d) were excluded. Core biopsy of the primary tumour was performed at diagnosis and repeated on day 21, immediately prior to the second cycle of chemotherapy. Six cycles of FEC chemotherapy (5-fluorouracil 600 mg/m^2^, epirubicin 60 mg/m^2^, and cyclophosphamide 600 mg/m^2^) were administered at 21-day intervals. Bi-dimensional clinical tumour measurements were recorded before every treatment. Four patients developed disease progression by clinical criteria during chemotherapy and proceeded to immediate surgery. The remaining women underwent breast-conserving surgery or mastectomy at the surgeon's discretion approximately 1 month after the final cycle of chemotherapy. All patients who were treated by breast-conserving surgery received post-operative radiation to the residual breast (40 Gy in 15 daily fractions plus 10-Gy boost to tumour bed in five fractions; *n *= 12) plus or minus lymph nodes (50 Gy in 25 fractions for a period of 5 weeks; *n *= 2). Post-mastectomy chest wall radiation was delivered to 13 of 15 patients (11 chest wall only, 2 chest wall and nodes). No patient received post-operative chemotherapy. Women with oestrogen receptor (ER)-positive tumours received 5 years of adjuvant tamoxifen (20 mg daily) starting after surgery.

The study was approved by the Luton & Dunstable NHS (National Health Service) Trust Ethics Committee and conducted in accordance with the Helsinki Declaration.

### Assessment of response

#### Clinical response

Standard UICC (International Union Against Cancer) criteria were used to define objective clinical response [9]. Changes in the calculated product of bi-dimensional tumour measurements on two successive evaluations were recorded at each visit. Complete response (CR) was defined as no residual palpable abnormality, partial response (PR) as greater than 50% tumour shrinkage, stable disease (SD) as less than 50% tumour shrinkage or no change, and progressive disease as an increase of at least 25%.

#### Pathological response

Although many different systems for grading pathological response have been proposed [10-15], no standard method for pathological assessment after chemotherapy has been adopted. A previously described simple scoring system that can be applied in clinical practice was therefore employed [16]. A consultant histopathologist (P.I. Richman) blinded to clinical outcome reviewed all paired biopsy and surgical specimens. We defined 'histological tumour response' by both (a) an apparent reduction in tumour cell/stroma ratio and (b) one or more chemotherapy-induced cytological changes (that is, enlarged cells with finely vacuolated cytoplasm, an enlarged vesicular nucleus with a prominent single eosinophilic nucleolus, or an enlarged hyperchromatic dense nucleus with an irregular outline). The following classification was used to score surgical specimens for pathological response: CR, no residual invasive carcinoma; PR, residual invasive carcinoma with histological tumour response; and SD, residual invasive carcinoma with no histological tumour response.

### Immunohistochemical technique

Four-micrometer sections were dried overnight at 37°C. Prior to antibody staining, the slides were pre-treated with microwave irradiation to unmask binding epitopes. After blocking of endogenous peroxidase activity with a 3% solution of hydrogen peroxide in methanol for 30 minutes, slides were immersed in 200 ml of 10 mM citric acid (pH 6.0) for 4 minutes on high power (800 W). After topping up of the buffer with distilled water, this step was repeated. The slides were then left to stand for 10 minutes in buffer at room temperature before being washed thoroughly in tap water. After three washes in tris-buffered saline (TBS), the slides were incubated with a 1:200 dilution of rabbit anti-Ki-67 polyclonal antibody (A0047; Dako UK Ltd., Cambridgeshire, UK) in TBS for 1–2 hours at room temperature. After three more washes in TBS, biotinylated goat anti-rabbit (Ki-67) in TBS was applied for 1 hour at room temperature. After an additional three washes, ABC complex (K0355; Dako UK Ltd.) was added for 1 hour at room temperature. The staining was visualised by adding diaminobenzidine (DAB kit SK 4100; Vector Laboratories, Burlington, CA, USA) for 5 minutes at room temperature. The slides were washed well in tap water and counterstained with Mayers haematoxylin for 10 seconds to 1 minute and then dehydrated, cleared, and mounted in distrene plasticiser xylene (DPX). Positive and negative controls were performed with each batch of slides. Paired core biopsies and surgical specimens from the same patient were stained on the same run.

Apoptotic cells were visualised using a commercial end labeling (TUNEL) assay previously described [8]. Briefly, endogenous peroxidase activity was inactivated with 1% hydrogen peroxide in phosphate-buffered saline (PBS) (pH 7.4) for 10 minutes. Nuclei of tissue sections were stripped of proteins by incubation with 0.5% pepsin (pH 2.0) (Sigma Chemical Co, Poole, Dorset, UK) for 30 minutes at 37°C. The sections were washed five times in distilled water to remove all traces of pepsin. Each section undergoing the TUNEL protocol was incubated for 5 minutes in Tris buffer (pH 7.6) and then for 1 hour at 37°C in 100 μl of reaction mixture consisting of 15 units TdT FPLC pure (Pharmacia, Windsor, Berkshire, UK), 0.5 nmol biotin-16-dUTP (Boehringer Mannheim, Mannheim, Germany), 5 mM cobalt chloride, 0.2 M sodium cacodylate, 25 mM Tris HCl (pH 6.6), and 0.25 mg/ml bovine serum albumin (BSA) dissolved in distilled water. After extensive washing in distilled water, the sections were incubated for 30 minutes at room temperature in 1:400 dilution of horseradish peroxidase conjugated to streptavidin (Dako UK Ltd.) in PBS supplemented with 1% BSA and 0.5% Tween 20. Colour was developed for 10 minutes using 0.05% diaminobenzidine plus 0.07% imidazole plus 0.1% hydrogen peroxide and further intensified in 0.5% copper sulphate with 0.9% sodium chloride for 1 minute. The sections were counterstained in Mayers haematoxylin, dehydrated, cleared in xylene, and mounted in DPX.

### Scoring methods

Immunohistochemical scoring was performed without prior knowledge of the clinical response. Ki-67 score was counted on a minimum of 10 randomly selected ×40 high-power fields containing representative sections of tumour and calculated as the percentage of positively stained cells to total cells. The AI was assessed by counting at least 3,000 malignant cells at ×400 magnification. Stained apoptotic cells were recorded, and cells displaying classic apoptotic morphology but not staining were also incorporated in the AI. Non-staining apoptotic cells were recognised in the midst of cells with normal morphology by having either condensed, irregular nuclei frequently with a crescent-shaped appearance or fragmented nuclei within cells showing cytoplasmic withdrawal. Areas with extensive necrosis were avoided.

### Statistical methods

Statistical analysis was carried out using JMP version 5.0 (SAS Institute Inc., Cary, NC, USA). Associations between ordinal variables were assessed using χ^2 ^analyses or the Fisher exact test in the case of two-by-two variables. Analyses involving Ki-67 and AI as continuous variables were investigated using analysis of variance. A logistic regression analysis was performed.

## Results

### Day-21 biopsy

Sufficient invasive carcinoma suitable for immunohistochemical analysis was present in 27 of the 39 day-21 biopsies. The remaining 12 patients were excluded from the analysis: eight yielded no demonstrable invasive tumour on day-21 biopsy, two comprised high-grade DCIS (ductal carcinoma *in situ*) only (presumably due to geographical miss), and two contained tiny foci of invasive tumour deemed too small to reliably interpret immunohistochemical staining.

### Patient demographics

Of the 27 evaluable patients, 52% were pre-menopausal. Most tumours were grade 2 (33%) or 3 (41%), stage T2 (44%) or T3 (42%), and clinically node-negative (63%) before treatment. Fifty-six percent were ER-positive and 41% HER-2 (human epidermal growth factor-2)-positive. The patient characteristics are shown in Table 1.

### Response rates

All 27 patients were evaluable for clinical response on completion of chemotherapy. Surgical blocks were retrieved for pathological scoring in 26 cases. The objective clinical response rate (CR + PR) was 56% (15/27). Four patients (15%) progressed by clinical criteria after two, two, four, and six cycles of chemotherapy, respectively, and proceeded to immediate surgery. The remaining eight patients (30%) had clinically stable disease on completion. Eight patients (30%) achieved a pathological response by histological criteria. There were no complete pathological responders, although two women had a 'near-pCR' with residual foci of invasive carcinoma measuring 1 and 2 mm in maximum dimension, respectively.

### Biological markers before, during, and after chemotherapy

The median and range of Ki-67 indices before chemotherapy, at day 21, and after treatment were 27.9% (4.1%–43.9%), 17.3% (4.1%–44.8%), and 21.7% (2.4%–50.4%), respectively. The apoptotic indices at baseline, day 21, and surgery were 1.92% (0.23%–5.4%), 1.69% (0.33%–11.2%), and 2.19% (0.9%–4.9%), respectively. At each time point, there was a significant positive relationship between these two parameters: the correlation coefficients were 0.47 (*p *= 0.026), 0.65 (*p *= 0.0005), and 0.66 (*p *= 0.0014) in the biopsy, day-21 and surgery samples, respectively.

### Changes in biological markers during and after chemotherapy

**Figure 1:**
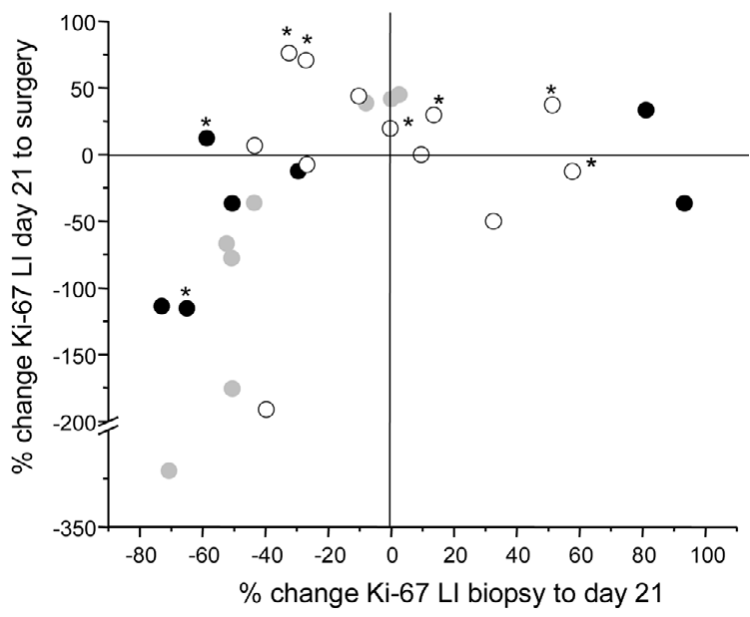
Changes in Ki-67 LI during treatment and clinical and pathological response. The data are expressed as % change between initial biopsy and day 21 relative to the initial biopsy score (x-axis) versus % change between day 21 and surgery relative to the day 21 index. (●) represents patients with a complete clinical response, (●) with a partial response and (○) represents no response. The asterisks represent those patients who achieved a pathological response.

A reduction in Ki-67 index from pre-treatment values was observed in 63% (17/27) of patients at day 21 and 69% (18/26) at surgery (Figure 1); there was no tumour material available for one patient at surgery. Eleven patients demonstrated sequential reductions in Ki-67 LI throughout the two study periods, and four patients showed sequential increases during therapy. Four of the 17 tumours that showed a reduction in LI between biopsy and day 21 showed increases in proliferation between day 21 and surgery. Of the 10 tumours that showed no change or an increase in Ki-67 LI during the first 3 weeks of chemotherapy, half displayed a subsequent reduction between day 21 and surgery.

**Figure 2:**
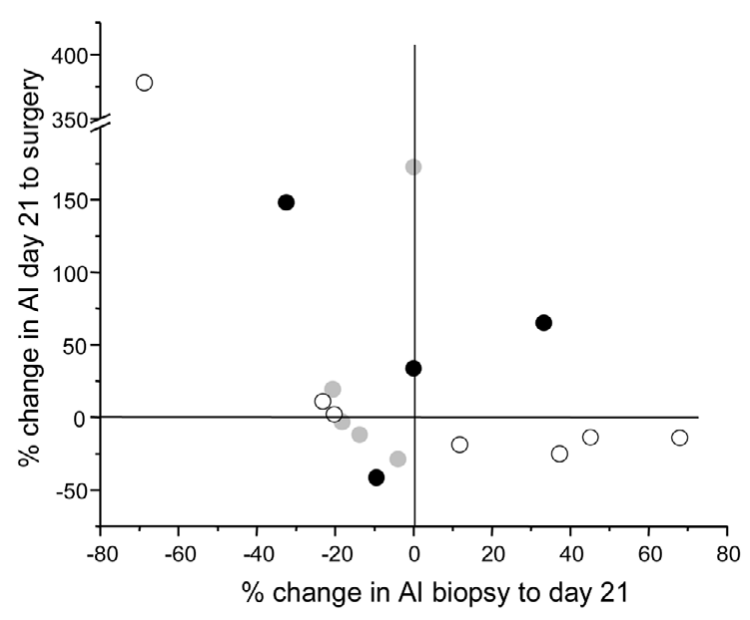
Changes in apoptotic LI during treatment and clinical and pathological response. The data are expressed as % change between initial biopsy and day 21 relative to the initial biopsy score (x-axis) versus % change between day 21 and surgery relative to the day 21 index. (●) represents patients with a complete clinical response, (●) with a partial response and (○) represents no response. The asterisks represent those patients who achieved a pathological response.

The AI was more difficult to assess in this material. There were seven instances in the day-21 biopsies and nine in the surgical material in which it was not possible to make a reliable measurement with the TUNEL assay. In those cases that were evaluable, there was a wide variation in percentage change in AI at day 21 compared with pre-treatment levels. AI decreased in 50% (10/20), increased in 45% (9/20), and was unchanged in one patient (Figure 2). Overall, between initial biopsy and surgery, a similar pattern was seen with eight (47%) out of 17 patients, with successful staining showing a reduction in AI. Between day 21 and surgery, the majority of tumours (10 of 18) increased in apoptotic activity (Figure 2). Unlike in the Ki-67 LI data, there was no consistent pattern in apoptosis throughout treatment.

### Ki-67 and clinical and pathological response

Neither pre-treatment nor post-chemotherapy median Ki-67 index differed significantly between clinical or pathological responders and non-responders. Clinical responders (CR+PR) had significantly lower median Ki-67 indices at day 21 than did non-responders (11.4% versus 27.0%, *p *= 0.02). A similar trend for lower day-21 Ki-67 in patients achieving a cCR was also recorded (*p *= 0.10). Clinical responders exhibited significantly greater percentage reductions in Ki-67 at day 21 than did non-responders (-50.6% versus -5.3%, *p *= 0.04). A decrease or no change in day-21 Ki-67 was observed in 80% (12/15) of clinical responders compared with 58% (7/12) of non-responders (Figure 1). In the 11 patients who showed sequential reductions in Ki-67 throughout the study period, nine (82%) achieved a clinical response (*p *= 0.019) (Figure 1).

Paradoxically, the median day-21 Ki-67 was higher in pathological responders (30.3% versus 14.1%, *p *= 0.046). There were no association between pathological response and changes in Ki-67 throughout the study period and no correlation between clinical and pathological responses (Figure 1).

### AI and clinical and pathological response

Median AI at all three time points and relative changes at day 21 and surgery did not differ significantly between clinical and pathological responders or non-responders (Figure 2). However, there was a trend toward higher pre-treatment AI in pathological responders (2.72 versus 1.65, *p *= 0.10). A non-significant trend toward increased apoptosis at day 21 in pathological responders was also observed (5.30 versus 1.68, *p *= 0.12). No pattern in the distribution of changes in day-21 AI emerges between clinical and pathological responders when the data are represented graphically.

### Biological characteristics of complete or 'near-complete' pathological responders

The ability to predict pCR, arguably the most useful endpoint of all, could not be assessed in this cohort, because no patient achieved a pCR. However, two patients had only tiny foci of residual invasive carcinoma demonstrable after chemotherapy. Both these 'near-pCR' patients had a very high AI at operation (3.96 and 3.61), significantly greater than patients not achieving a 'near-pCR' (*p *= 0.04). One of these two patients was evaluable for day-21 AI; a large increase in AI was seen (5.3 versus 3.86) after the first cycle of chemotherapy. No clear trend in changes in Ki-67 during or after treatment was seen in the two patients with excellent pathological tumour regression.

### Logistic regression analysis for prediction of response by different modalities of assessment

Logistic regression analyses were performed to establish which, if any, of the biological marker variables measured at different time points could predict response outcomes by clinical, radiological, or pathological criteria (Table 2). Increased AI at day 21 was a statistically significant predictor of pathological response (*p *= 0.049). Similarly, greater Ki-67 indices at day 21 and higher AI at surgery displayed a strong trend for predicting pathological response (*p *= 0.06 and 0.06, respectively). Reductions in Ki-67 and AI at day 21 were strongly predictive of better clinical response by UICC category (*p *= 0.01 and 0.02, respectively). No significant associations were observed between the various biological markers and clinical CR or radiological response assessed by mammography and/or ultrasound. Low baseline AI was associated with poor worst radiological response (*p *= 0.04).

## Discussion

The prognostic significance of pre-treatment Ki-67 index in breast tumours varies. Intuitively, rapidly proliferating tumours confer a poor prognosis, and the majority of studies confirm this association [17-26]. In some series, breast tumours with a high proliferative index have a worse prognosis despite endocrine treatment [27,28] or chemotherapy [29]. However, other authors report no significant difference in outcome after chemotherapy or hormone treatment in patients with rapidly proliferating tumours compared with those with more slowly growing tumours [30-33].

Changes in tumour cell proliferation before and after pre-operative treatment have also been evaluated. A reduction in Ki-67 index has been demonstrated after chemotherapy [30,34,35], tamoxifen therapy [31,36], and chemoendocrine therapy [37,38]. More recently, studies have focused on the evaluation of early changes in cell proliferation during treatment by analysing Ki-67 index in repeat tumour samples taken at varying intervals during chemotherapy. Two studies at the Royal Marsden Hospital (London, UK) performed on cytological material obtained from fine needle aspiration (FNA) during chemoendocrine treatment showed that reductions in Ki-67 proliferation index after 10, 14, or 21 days significantly predict clinical response [37,38]. However, Billgren and colleagues demonstrated that a decrease of more than 25% in proliferating fraction after the first course of chemotherapy significantly predicted a reduced risk of disease recurrence (*p *= 0.033) but showed no correlation with local objective response [39]. Multivariate analysis revealed that the decrease in proliferating fraction significantly added prognostic information to lymph node status. In a similar study, patients who responded to neoadjuvant chemotherapy and concurrent tamoxifen were found to be more likely to have a reduction in Ki-67 ten days after chemotherapy than were non-responders [40]. Post-treatment Ki-67 index is also of prognostic importance: In a series of 42 patients treated with primary chemotherapy, high proliferative index in residual tumour was associated with a worse disease-free survival [41].

In this study, there was no significant difference in baseline Ki-67 or AI between responders and non-responders assessed by clinical, pathological, or radiological criteria. Both pre-treatment and post-chemotherapy cell proliferation and apoptosis failed to predict response by any modality of assessment.

More than two thirds of tumours exhibited a decrease from baseline Ki-67 index at day 21 and at surgery. There was no significant difference in the magnitude of the decrease in Ki-67 from baseline to surgery between different groups. The degree of cell proliferation measured before or after chemotherapy was not able to discriminate clinical or pathological responders from non-responders. Clinical responders were more likely to exhibit a reduction in Ki-67 index after one cycle of chemotherapy. This group also displayed larger relative decreases in cell proliferation after the first cycle of treatment (median -50.6, range -73.0 to 93.3) than did non-responders (median -5.3, range -43.4 to 57.7) (*p *= 0.04). Paradoxically, Ki-67 expression at day 21 was greater in pathological responders compared with non-responders, despite the fact that the distribution of pre-treatment Ki-67 LI was similar in both groups. This observation seems counterintuitive because tumour regression would be expected to be accompanied by a reduction in cell proliferation. However, there was no association between clinical response and those patients who achieved a partial pathological response. These findings underline the uncertainty surrounding the optimum method of assessment of response in biomarker studies and raise concerns that one (or perhaps both) of the classifications of response used in this study may not be a reliable surrogate endpoint.

More than half the patients showed an increase in measured cell proliferation between day 21 and surgery. In responding patients, the reduction in Ki-67 index after one cycle of treatment was not sustained and was often followed by a rebound increase in cell proliferation by the time of surgery (responders 26.8, range 2.4 to 48.0; non-responders 18.9, range 6.8 to 50.4).

The observed changes in proliferation during treatment may have implications for determining the optimum duration of neoadjuvant chemotherapy prior to surgery for operable primary breast cancer. Recently published randomised clinical trials suggest that the addition of sequential taxane chemotherapy after four cycles of anthracycline PST increases clinical and pathological response rates and translates into improved overall survival [15,42]. The rebound increases in cell proliferation noted after six cycles of anthracycline treatment in this study may partly explain the superior clinical results achieved when patients are switched to non-cross-resistant chemotherapy regimens midway through neoadjuvant treatment. This phenomenon warrants further investigation to establish whether changes in tumour cell kinetics during treatment can identify which patients are most likely to benefit from sequential chemotherapy schedules.

Wide variations in AI were seen both during and after chemotherapy. The non-significant trend toward increased AI in pathological responders at day 21 was confirmed by the logistic regression analysis showing that increased day-21 AI is a statistically significant predictor of pathological response. This observation suggests that tumours exhibiting high levels of cell death after one cycle of chemotherapy are more likely to achieve pathological regression. The high AI seen in the two near-pCR patients at operation indicates that increased apoptosis after chemotherapy may also predict which patients will have a good pathological response. Analysis of a larger cohort is required to explore this hypothesis further. The magnitude of changes in AI during treatment did not predict clinical, radiological, or pathological response to treatment.

The optimum time point for detecting early cell kinetic changes that may predict clinical and pathological outcomes is unknown. Other groups have repeated FNA cytology 10 days after chemoendocrine treatment [37,38]. Day 21 was arbitrarily chosen as a convenient time in this study, to coincide with patients' return to hospital for their second cycle of chemotherapy, although there are no convincing data that it is the most appropriate time to test biomarkers. It is possible that 21 days after chemotherapy is too late to observe the peaks of apoptotic response and suppression of proliferation induced by cytotoxic treatment; there may be earlier times when the biologic response to treatment is more critically related to therapeutic outcome. Indeed, there is some evidence that apoptotic response after chemotherapy lasts for several days only [43-45]. Ideally, serial biopsies may help to chart the precise pattern of changes in biological markers during treatment; realistically, however, large studies of this type are impractical, because few patients are likely to agree to repeated invasive tumour biopsies.

It is important to recognise the potential limitations of this study. Like most published series in this field, the number of patients reported is small. The use of tumour biopsies to assess molecular marker expression before and after treatment has become increasingly widespread as the search for predictive markers for neoadjuvant chemotherapy continues. Critics initially argued that this approach was subject to sampling error and intra-tumour variability. However, the widely quoted validation study by Ellis and colleagues [35] demonstrated that core biopsies accurately reflect the expression of biological markers in whole tumour sections.

In addition to clinical response, a novel descriptive histological response analysis was used to grade pathological response in this study. Although this system has not been prospectively validated or proven to relate directly to survival, the strong body of evidence that pCR is a good prognostic indicator for long-term survival justifies its use. Unfortunately, the analysis was hampered by the absence of complete pathological responders in this small series, forcing the authors to adopt the more widely used assessment of clinical response as an endpoint.

## Conclusion

In this small study, pre-treatment or post-chemotherapy median Ki-67 index, median AI at all three time points, and relative changes at day 21 and surgery did not differ significantly between clinical or pathological responders and non-responders. Clinical responders achieved significantly greater percentage reductions in Ki-67 and lower median Ki-67 indices at day 21 than did non-responders. Pathological responders displayed higher median day-21 Ki-67 expression. Increased day-21 AI was a statistically significant predictor of pathological response. A strong trend for predicting pathological response was seen with higher Ki-67 indices at day 21 and AI at surgery.

The clinical utility of early changes in biological marker expression during chemotherapy remains unclear. For the time being, clinical decision-making should not be based upon individual biological tumour marker profiles until further prospectively validated evidence confirming the reliability of predictive markers is available. In the meantime, large prospective clinical trials of neoadjuvant chemotherapy should include parallel biological marker studies to facilitate immunohistochemistry and microarray analysis on histological tissue taken at various time points before, during, and after neoadjuvant chemotherapy to continue the search for clinically useful predictive biomarkers.

## Abbreviations

AI = apoptotic index; BSA = bovine serum albumin; cCR = complete clinical response; CR = complete response; DPX = distrene plasticiser xylene; ER = oestrogen receptor; FEC = 5-fluorouracil, epirubicin, and cyclophosphamide; FNA = fine needle aspiration; LI = labeling index (Ki-67 index); PBS = phosphate-buffered saline; pCR = complete pathological response; PR = partial response; PST = primary systemic therapy; SD = stable disease; TBS = tris-buffered saline; TUNEL = terminal transferase uridyl nick-end labeling; UICC = International Union Against Cancer.

## Competing interests

The authors declare that they have no competing interests.

## Authors' contributions

RJB performed core biopsies, collected tissue samples, carried out immunohistochemical staining and scoring, and drafted the manuscript. GDW assisted with statistical analysis and helped write the manuscript. MD helped perform TUNEL assays and gave advice on manuscript content. IK performed statistical analyses. PIR scored samples for pathological response. FD assisted with immunohistochemical staining. SD performed TUNEL assays. AM conceived the study, participated in its design and coordination, and helped finalise the manuscript. All authors read and approved the final manuscript.

## Figures and Tables

**Table 1:** Patient characteristics

		***n***	**Percentage**
Age: median		51 years	
Age: range		29–65	
Menstrual status	Pre	14	52
	Peri	6	22
	Post	7	26
Clinical TNM stage at diagnosis	T2	12	44
	T3	13	48
	T4	2	7
Clinical node status	N0	17	63
	N1	10	37
Breast	Right	15	56
	Left	12	44
Breast surgery	Wide excision	12	44
	Mastectomy	15	56
Postoperative radiotherapy	Nil	2	7
	Breast	12	44
	Chest wall	13	48
ER status (biopsy)	ER-positive	15	56
	ER-negative	12	44
HER-2 status (biopsy)	HER-2-positive	11	41
	HER-2-negative	16	59
Tumour grade	Unknown*	5	19
	G1	2	7
	G2	9	33
	G3	11	41
Pathological T stage	pCR	0	0
	pT1	8	30
	pT2	17	63
	pT3	2	7
Pathological N stage	pN0	11	41
	pN1	12	44
	pNX	4	15

*Grading not possible due to chemotherapy artefact. ER, oestrogen receptor; HER-2, human epidermal growth factor 2; TNM, tumour, node, metastasis.

**Table 2:** Logistic regression analysis showing significant associations for prediction of response by different modalities of assessment and response classifications

**Response variable**	***p *value**
**Pathological response (R/NR)**	
Ki-67 D21	0.0616
AI D21	0.0497
AI Sx	0.0620
**Pathological CR**	
Not assessable	-
**'Near' pathological CR**	
No significant associations	-
**Clinical response (CR/PR/SD/PD)**	
*[Path T stage T1 versus T3*	*0.0028]*
Ki-67 D21	0.0097
AI D21	0.0224
**Clinical response (CR/PR/NR)**	
*[Path T stage T1 versus T3*	*0.0066]*
Ki-67 D21	0.0326
AI D21	0.0224
**Clinical response (R/NR)**	
Ki-67 D21	0.0323
**Clinical CR**	
No significant associations	-
**Radiological response (R/NR)**	-
No significant associations	
**Mammographic response (CR/PR/SD/PD)**	
No significant associations	-
**USS response (CR/PR/SD/PD)**	
No significant associations	-
**Worst radiological response (CR/PR/SD/PD)**	
AI biopsy	0.0418
**Worst radiological response (CR/PR/NR)**	
No significant associations	-

AI, apoptotic index; CR, complete response;D21, day-21; NR, non-responder; PD, progressive disease; PR, partial response; R, responder; SD, stable disease; USS, ultrasound scan.

## References

[B1] Makris A, Powles TJ, Ashley SE, Chang J, Hickish T, Tidy VA, Nash AG, Ford HT (1998). A reduction in the requirements for mastectomy in a randomized trial of neoadjuvant chemoendocrine therapy in primary breast cancer. Ann Oncol.

[B2] Fisher B, Brown A, Mamounas E, Wieand S, Robidoux A, Margolese RG, Cruz AB, Fisher ER, Wickerham DL, Wolmark N (1997). Effect of preoperative chemotherapy on local-regional disease in women with operable breast cancer: findings from National Surgical Adjuvant Breast and Bowel Project B-18. J Clin Oncol.

[B3] Fisher B, Bryant J, Wolmark N, Mamounas E, Brown A, Fisher ER, Wickerham DL, Begovic M, DeCillis A, Robidoux A (1998). Effect of preoperative chemotherapy on the outcome of women with operable breast cancer. J Clin Oncol.

[B4] Cleator SJ, Makris A, Ashley SE, Lal R, Powles TJ (2005). Good clinical response of breast cancers to neoadjuvant chemoendocrine therapy is associated with improved overall survival. Ann Oncol.

[B5] Gasparini G, Boracchi P, Verderio P, Bevilacqua P (1994). Cell kinetics in human breast cancer: comparison between the prognostic value of the cytofluorimetric S-phase fraction and that of the antibodies to Ki-67 and PCNA antigens detected by immunocytochemistry. Int J Cancer.

[B6] Dawson AE, Norton JA, Weinberg DS (1990). Comparative assessment of proliferation and DNA content in breast carcinoma by image analysis and flow cytometry. Am J Pathol.

[B7] Vielh P, Chevillard S, Mosseri V, Donatini B, Magdelenat H (1990). Ki67 index and S-phase fraction in human breast carcinomas. Comparison and correlations with prognostic factors. Am J Clin Pathol.

[B8] Mainwaring PN, Ellis PA, Detre S, Smith IE, Dowsett M (1998). Comparison of *in situ* methods to assess DNA cleavage in apoptotic cells in patients with breast cancer. J Clin Pathol.

[B9] Hayward JL, Carbone PP, Heuson JC, Kumaoka S, Segaloff A, Rubens RD (1977). Assessment of response to therapy in advanced breast cancer: a project of the Programme on Clinical Oncology of the International Union Against Cancer, Geneva, Switzerland. Cancer.

[B10] Sataloff DM, Mason BA, Prestipino AJ, Seinige UL, Lieber CP, Baloch Z (1995). Pathologic response to induction chemotherapy in locally advanced carcinoma of the breast: a determinant of outcome. J Am Coll Surg.

[B11] Akashi-Tanaka S, Tsuda H, Fukuda H, Watanabe T, Fukutomi T (1996). Prognostic value of histopathological therapeutic effects and mitotic index in locally advanced breast cancers after neoadjuvant chemotherapy. Jpn J Clin Oncol.

[B12] Chevallier B, Roche H, Olivier JP, Chollet P, Hurteloup P (1993). Inflammatory breast cancer. Pilot study of intensive induction chemotherapy (FEC-HD) results in a high histologic response rate. Am J Clin Oncol.

[B13] Honkoop AH, Pinedo HM, De Jong JS, Verheul HM, Linn SC, Hoekman K, Wagstaff J, van Diest PJ (1997). Effects of chemotherapy on pathologic and biologic characteristics of locally advanced breast cancer. Am J Clin Pathol.

[B14] Kuerer HM, Newman LA, Buzdar AU, Dhingra K, Hunt KK, Buchholz TA, Binkley SM, Strom EA, Ames FC, Ross MI (1998). Pathologic tumor response in the breast following neoadjuvant chemotherapy predicts axillary lymph node status. Cancer J Sci Am.

[B15] Smith IC, Heys SD, Hutcheon AW, Miller ID, Payne S, Gilbert FJ, Ah-See AK, Eremin O, Walker LG, Sarkar TK (2002). Neoadjuvant chemotherapy in breast cancer: significantly enhanced response with docetaxel. J Clin Oncol.

[B16] Burcombe RJ, Makris A, Richman PI, Daley FM, Noble S, Pittam M, Wright D, Allen SA, Dove J, Wilson GD (2005). Evaluation of ER, PgR, HER-2 and Ki-67 as predictors of response to neoadjuvant anthracycline chemotherapy for operable breast cancer. Br J Cancer.

[B17] Brown RW, Allred CD, Clark GM, Osborne CK, Hilsenbeck SG (1996). Prognostic value of Ki-67 compared to S-phase fraction in axillary node-negative breast cancer. Clin Cancer Res.

[B18] Gaglia P, Bernardi A, Venesio T, Caldarola B, Lauro D, Cappa AP, Calderini P, Liscia DS (1993). Cell proliferation of breast cancer evaluated by anti-BrdU and anti-Ki-67 antibodies: its prognostic value on short-term recurrences. Eur J Cancer.

[B19] Gottardi O, Tabiadon D, Scanzi F, Bono A, Majno M, Ferrari M, Colombo P (1992). Clinical and prognostic usefulness of Ki67 determination in breast carcinoma. Pathologica.

[B20] Lee AK, Loda M, Mackarem G, Bosari S, DeLellis RA, Heatley GJ, Hughes K (1997). Lymph node negative invasive breast carcinoma 1 centimeter or less in size (T1a, bNOMO): clinicopathologic features and outcome. Cancer.

[B21] Pierga JY, Leroyer A, Viehl P, Mosseri V, Chevillard S, Magdelenat H (1996). Long term prognostic value of growth fraction determination by Ki-67 immunostaining in primary operable breast cancer. Breast Cancer Res Treat.

[B22] Pinder SE, Wencyk P, Sibbering DM, Bell JA, Elston CW, Nicholson R, Robertson JF, Blamey RW, Ellis IO (1995). Assessment of the new proliferation marker MIB1 in breast carcinoma using image analysis: associations with other prognostic factors and survival. Br J Cancer.

[B23] Railo M, Lundin J, Haglund C, von Smitten K, von Boguslawsky K, Nordling S (1997). Ki-67, p53, Er-receptors, ploidy and S-phase as prognostic factors in T1 node negative breast cancer. Acta Oncol.

[B24] Railo M, Nordling S, von Boguslawsky K, Leivonen M, Kyllonen L, von Smitten K (1993). Prognostic value of Ki-67 immunolabelling in primary operable breast cancer. Br J Cancer.

[B25] Veronese SM, Gambacorta M, Gottardi O, Scanzi F, Ferrari M, Lampertico P (1993). Proliferation index as a prognostic marker in breast cancer. Cancer.

[B26] Wintzer HO, Zipfel I, Schulte-Monting J, Hellerich U, von Kleist S (1991). Ki-67 immunostaining in human breast tumors and its relationship to prognosis. Cancer.

[B27] Archer SG, Eliopoulos A, Spandidos D, Barnes D, Ellis IO, Blamey RW, Nicholson RI, Robertson JF (1995). Expression of ras p21, p53 and c-erbB-2 in advanced breast cancer and response to first line hormonal therapy. Br J Cancer.

[B28] Daidone MG, Luisi A, Martelli G, Benini E, Veneroni S, Tomasic G, De Palo G, Silvestrini R (2000). Biomarkers and outcome after tamoxifen treatment in node-positive breast cancers from elderly women. Br J Cancer.

[B29] Clahsen PC, van de Velde CJ, Duval C, Pallud C, Mandard AM, Delobelle-Deroide A, van den Broek L, Sahmoud TM, van de Vijver MJ (1998). p53 protein accumulation and response to adjuvant chemotherapy in premenopausal women with node-negative early breast cancer. J Clin Oncol.

[B30] Bottini A, Berruti A, Bersiga A, Brizzi MP, Bruzzi P, Aguggini S, Brunelli A, Bolsi G, Allevi G, Generali D (2001). Relationship between tumour shrinkage and reduction in Ki67 expression after primary chemotherapy in human breast cancer. Br J Cancer.

[B31] Clarke RB, Laidlaw IJ, Jones LJ, Howell A, Anderson E (1993). Effect of tamoxifen on Ki67 labelling index in human breast tumours and its relationship to oestrogen and progesterone receptor status. Br J Cancer.

[B32] MacGrogan G, Mauriac L, Durand M, Bonichon F, Trojani M, de Mascarel I, Coindre JM (1996). Primary chemotherapy in breast invasive carcinoma: predictive value of the immunohistochemical detection of hormonal receptors, p53, c-erbB-2, MiB1, pS2 and GST pi. Br J Cancer.

[B33] Rudas M, Gnant MF, Mittlbock M, Neumayer R, Kummer A, Jakesz R, Reiner G, Reiner A (1994). Thymidine labeling index and Ki-67 growth fraction in breast cancer: comparison and correlation with prognosis. Breast Cancer Res Treat.

[B34] Bottini A, Berruti A, Bersiga A, Brunelli A, Brizzi MP, Marco BD, Cirillo F, Bolsi G, Bertoli G, Alquati P, Dogliotti L (1996). Effect of neoadjuvant chemotherapy on Ki67 labelling index, c-erbB-2 expression and steroid hormone receptor status in human breast tumours. Anticancer Res.

[B35] Ellis PA, Smith IE, Detre S, Burton SA, Salter J, A'Hern R, Walsh G, Johnston SR, Dowsett M (1998). Reduced apoptosis and proliferation and increased Bcl-2 in residual breast cancer following preoperative chemotherapy. Breast Cancer Res Treat.

[B36] Dardes RD, Horiguchi J, Jordan VC (2000). A pilot study of the effects of short-term tamoxifen therapy on Ki-67 labelling index in women with primary breast cancer. Int J Oncol.

[B37] Makris A, Powles TJ, Allred DC, Ashley S, Ormerod MG, Titley JC, Dowsett M (1998). Changes in hormone receptors and proliferation markers in tamoxifen treated breast cancer patients and the relationship with response. Breast Cancer Res Treat.

[B38] Chang J, Powles TJ, Allred DC, Ashley SE, Clark GM, Makris A, Assersohn L, Gregory RK, Osborne CK, Dowsett M (1999). Biologic markers as predictors of clinical outcome from systemic therapy for primary operable breast cancer. J Clin Oncol.

[B39] Billgren AM, Rutqvist LE, Tani E, Wilking N, Fornander T, Skoog L (1999). Proliferating fraction during neoadjuvant chemotherapy of primary breast cancer in relation to objective local response and relapse-free survival. Acta Oncol.

[B40] Makris A, Powles TJ, Allred DC, Ashley SE, Trott PA, Ormerod MG, Titley JC, Dowsett M (1999). Quantitative changes in cytological molecular markers during primary medical treatment of breast cancer: a pilot study. Breast Cancer Res Treat.

[B41] Honkoop AH, van Diest PJ, de Jong JS, Linn SC, Giaccone G, Hoekman K, Wagstaff J, Pinedo HM (1998). Prognostic role of clinical, pathological and biological characteristics in patients with locally advanced breast cancer. Br J Cancer.

[B42] Bear HD, Anderson S, Brown A, Smith R, Mamounas EP, Fisher B, Margolese R, Theoret H, Soran A, Wickerham DL (2003). The effect on tumor response of adding sequential preoperative docetaxel to preoperative doxorubicin and cyclophosphamide: preliminary results from National Surgical Adjuvant Breast and Bowel Project Protocol B-27. J Clin Oncol.

[B43] Buchholz TA, Davis DW, McConkey DJ, Symmans WF, Valero V, Jhingran A, Tucker SL, Pusztai L, Cristofanilli M, Esteva FJ (2003). Chemotherapy-induced apoptosis and Bcl-2 levels correlate with breast cancer response to chemotherapy. Cancer J.

[B44] Symmans WF, Volm MD, Shapiro RL, Perkins AB, Kim AY, Demaria S, Yee HT, McMullen H, Oratz R, Klein P (2000). Paclitaxel-induced apoptosis and mitotic arrest assessed by serial fine-needle aspiration: implications for early prediction of breast cancer response to neoadjuvant treatment. Clin Cancer Res.

[B45] Chang J, Ormerod M, Powles TJ, Allred DC, Ashley SE, Dowsett M (2000). Apoptosis and proliferation as predictors of chemotherapy response in patients with breast carcinoma. Cancer.

